# Supplementary material for: Challenges of the Implementation of a Delirium Rate Scale in a Pediatric Intensive Care Unit: A Qualitative Approach
Source: Healthcare (Basel). 2023 Dec 26;12(1):52. doi: 10.3390/healthcare12010052 (PMC10779040; doi:10.3390/healthcare12010052)
Supplement: Supplementary file 1 [file healthcare-12-00052-s001.zip › Supplementary Materials S3. English editing.pdf]

Supplementary Materials S3.

SEMI-STRUCTURED INDIVIDUAL INTERVIEW SCRIPT

General information

• How old are you?
• What is your level of professional training?
• How long have you been working in this unit?
• Have you worked in another unit before here? Which? How long?
• What is your current position?
• How many patients do you usually take care of during your work day?

Axis 1: The phenomenon of delirium in the Pediatric ICU.

• In your experience, does pediatric delirium exist in your unit? Do you consider it to be an important reality?

• In your experience, how many cases of delirium have you recently seen in your unit?
• According to your experience and perception, how many children suffer from this syndrome in your unit? At what point do you consider that delirium is given the most importance in your unit?

Axis 2: Tools to combat this phenomenon

• According to your experience, what elements help combat this phenomenon? Do you know this phenomenon and its determining factors in depth?
• In your experience, has the use of the CAPD detection tool helped to improve the impact of delirium on your unit?

Axis 3: Involvement as a professional in the unit.

• From your position as a nurse, do you feel involved as a professional who plays a key role in combating delirium in the unit? Is your opinion as a nurse taken into account in this regard?
• In your opinion, is it a specific task as a nurse to combat delirium in your unit, or is it a collateral duty?

Axis 4: Training and skills to combat delirium

• Do you have sufficient training in measures to combat delirium? Have you received training in the management of CAPD? Do you feel confident in your knowledge and skills to manage delirium care in your unit?

- **Do you want to add something else regarding the topic of delirium in your unit?**
